# Comparison of manual and artificial intelligence-automated choroidal thickness segmentation of optical coherence tomography imaging in myopic adults

**DOI:** 10.1186/s40662-024-00385-2

**Published:** 2024-06-03

**Authors:** Zhi Wei Lim, Jonathan Li, Damon Wong, Joey Chung, Angeline Toh, Jia Ling Lee, Crystal Lam, Maithily Balakrishnan, Audrey Chia, Jacqueline Chua, Michael Girard, Quan V. Hoang, Rachel Chong, Chee Wai Wong, Seang Mei Saw, Leopold Schmetterer, Noel Brennan, Marcus Ang

**Affiliations:** 1 Yong Loo Lin School of Medicine, National University of Singapore, Singapore, Singapore; 2 Department of Ophthalmology, University of California, San Francisco, CA USA; 3 Singapore Eye Research Institute, Singapore National Eye Centre, Singapore, Singapore; 4 Ophthalmology and Visual Sciences Department, Duke-NUS Medical School, Singapore, Singapore; 5 SERI-NTU Advanced Ocular Engineering (STANCE), Singapore Eye Research Institute and Nanyang Technological University, Singapore, Singapore; 6 Center for Medical Physics and Biomedical Engineering, Medical University Vienna, Vienna, Austria; 7 Duke-NUS Medical School, National University of Singapore, Singapore, Singapore; 8 Institute of Molecular and Clinical Ophthalmology, Basel, Switzerland; 9 Department of Ophthalmology, Yong Loo Lin School of Medicine, National University of Singapore, Singapore, Singapore; 10 Department of Ophthalmology, Edward S. Harkness Eye Institute, Columbia University Vagelos College of Physicians and Surgeons, New York, NY USA; 11 Saw Swee Hock School of Public Health, National University of Singapore, Singapore, Singapore; 12 School of Chemistry, Chemical Engineering and Biotechnology, Nanyang Technological University, Singapore, Singapore; 13 Department of Clinical Pharmacology, Medical University Vienna, Vienna, Austria; 14 Johnson & Johnson Vision, Jacksonville, FL USA

**Keywords:** Choroidal thickness, Myopia, Optical coherence tomography, Automated segmentation, Manual segmentation

## Abstract

**Background:**

Myopia affects 1.4 billion individuals worldwide. Notably, there is increasing evidence that choroidal thickness plays an important role in myopia and risk of developing myopia-related conditions. With the advancements in artificial intelligence (AI), choroidal thickness segmentation can now be automated, offering inherent advantages such as better repeatability, reduced grader variability, and less reliance for manpower. Hence, we aimed to evaluate the agreement between AI-automated and manual segmented measurements of subfoveal choroidal thickness (SFCT) using two swept-source optical coherence tomography (OCT) systems.

**Methods:**

Subjects aged ≥ 16 years, with myopia of ≥ 0.50 diopters in both eyes, were recruited from the Prospective Myopia Cohort Study in Singapore (PROMYSE). OCT scans were acquired using Triton DRI-OCT and PLEX Elite 9000. OCT images were segmented both automatically with an established SA-Net architecture and manually using a standard technique with adjudication by two independent graders. SFCT was subsequently determined based on the segmentation. The Bland–Altman plot and intraclass correlation coefficient (ICC) were used to evaluate the agreement.

**Results:**

A total of 229 subjects (456 eyes) with mean [± standard deviation (SD)] age of 34.1 (10.4) years were included. The overall SFCT (mean ± SD) based on manual segmentation was 216.9 ± 82.7 µm with Triton DRI-OCT and 239.3 ± 84.3 µm with PLEX Elite 9000. ICC values demonstrated excellent agreement between AI-automated and manual segmented SFCT measurements (PLEX Elite 9000: ICC = 0.937, 95% CI: 0.922 to 0.949, *P* < 0.001; Triton DRI-OCT: ICC = 0.887, 95% CI: 0.608 to 0.950, *P* < 0.001). For PLEX Elite 9000, manual segmented measurements were generally thicker when compared to AI-automated segmented measurements, with a fixed bias of 6.3 µm (95% CI: 3.8 to 8.9, *P* < 0.001) and proportional bias of 0.120 (*P* < 0.001). On the other hand, manual segmented measurements were comparatively thinner than AI-automated segmented measurements for Triton DRI-OCT, with a fixed bias of − 26.7 µm (95% CI: − 29.7 to − 23.7, *P* < 0.001) and proportional bias of − 0.090 (*P* < 0.001).

**Conclusion:**

We observed an excellent agreement in choroidal segmentation measurements when comparing manual with AI-automated techniques, using images from two SS-OCT systems. Given its edge over manual segmentation, automated segmentation may potentially emerge as the primary method of choroidal thickness measurement in the future.

**Supplementary Information:**

The online version contains supplementary material available at 10.1186/s40662-024-00385-2.

## Background

Globally, myopia affects 1.4 billion individuals, amounting to 23% of the world population [1]. Those suffering from high myopia (163 million individuals) are exposed to an increased risk of pathologic myopia and subsequent visual impairment [2, 3]. There is now increasing evidence that the choroid plays an important role in myopia and risk of developing pathology related to myopia [4–9]. Thinner choroids have been observed to be significantly associated with longer axial lengths, myopic spherical equivalent and more importantly, an increased risk of poorer best corrected visual acuity and myopic macular degeneration (MMD) [4–14]. Given its clinical significance, choroidal thickness has been employed as a biomarker for the staging of MMD [15].

Recently, imaging of the choroid has improved with newer optical coherence tomography (OCT) technologies, including the enhanced depth imaging spectral-domain OCT (EDI SD-OCT) and swept-source OCT (SS-OCT) [16, 17]. The SS-OCT utilizes a novel tuneable laser with increased operating wavelength that is capable of deeper tissue penetration with reduced light scattering and faster image acquisition, enabling enhanced tomography of the choroid [18, 19]. Given the vast array of imaging protocols and segmentation algorithms adopted across manufacturers, it is essential to determine the agreement between OCT machines, as this would facilitate clinical interpretation and comparison between inter-modality measurements [18, 20–27]. To the best of our knowledge, there have been no studies comparing choroidal thickness measurements between two established swept-source OCT modalities – Triton DRI-OCT and PLEX Elite 9000.

Furthermore, with the advancements in artificial intelligence (AI), choroidal thickness segmentation can now be automated [28–31]. As compared with manual segmentation, AI-automated segmentation possesses the intrinsic advantages of better repeatability with reduced inter- and intra-grader variability, and the need for less manpower. With these attributes, clinicians using AI-automated segmentation can be more certain of the significance of inter-visit changes detected in choroidal thickness and measurements may be more feasible to perform in day-to-day clinical setting. In this regard, Cahyo et al. recently introduced a novel multi-task learning approach – SA-Net, aimed to perform automated choroidal segmentation of 3-dimensional (3D) OCT images in the clinical setting [28]. Nonetheless, automated measurements derived using SA-Net have yet to be compared to manually segmented choroidal measurements.

Hence, the objective of our study is to evaluate the choroidal thickness in a population of myopic adults and ascertain the agreement in choroidal thickness measurements using a previously described AI-automated (SA-Net) technique versus a manual segmentation technique, in two different SS-OCT systems. In addition, we aim to evaluate the agreement in choroidal thickness between both SS-OCT modalities as a secondary objective of the study. Findings from this study will contribute towards utilizing choroidal thickness as a clinical biomarker in myopia and establishing meaningful comparisons between choroidal thickness measured with different OCT modalities.

## Methods

### Study population

We conducted a cross-sectional study utilizing subjects recruited from the ongoing Prospective Myopia Cohort Study in Singapore (PROMYSE). In brief, subjects aged 16 years and over, with myopia of ≥ 0.50 diopters in both eyes, were recruited from the Singapore National Eye Centre (SNEC) from July 2019 to May 2022. For this study, we included subjects with OCT assessments from both Triton DRI-OCT and PLEX Elite 9000. Subjects with poor OCT scan quality or ocular diseases which may impede the accuracy of OCT acquisition, such as significant corneal opacities, advanced cataracts, vitreous opacities, retinal detachment, retinal dystrophies, macular oedema, and macular scarring, were excluded. All study procedures adhered to the principles of the Declaration of Helsinki. Ethics approval was obtained from the Centralized Institutional Review Board of Singapore Health Services (CIRB Reference Number: 2019/2069). Written informed consent was obtained from all subjects.

### Ophthalmic assessment

All subjects underwent comprehensive ophthalmic examinations at SNEC. Presenting visual acuity was measured using the logarithm of the minimum angle of resolution (LogMAR) chart (Lighthouse International, New York, NY, USA). Manifest refraction was subsequently performed by certified research optometrists and spherical equivalent was determined as the sum of spherical power and half of cylindrical power. Anterior and posterior segment examinations were performed by ophthalmologists using slit lamp biomicroscopy after pupillary dilation as specified below. Axial length was measured using IOL Master (Carl Zeiss Meditec, AG, Jena, Germany). An axial length of ≥ 26 mm was defined as high myopia [32]. Fundus photographs and SS-OCT scans were acquired following pupillary dilation with the administration of two drops of tropicamide 1%, five minutes apart. Myopia-related retinal morphological changes were documented, namely peripapillary atrophy (PPA), disc tilt, MMD and MMD-plus [based on the meta-analyses of pathologic myopia study (META-PM)] [33], macular hole, myopic tractional maculopathy (MTM), peripheral retinal degeneration, retinoschisis, posterior staphyloma, epiretinal membrane (ERM), dome shaped macula (DSM), and intrachoroidal cavitation. MMD consisted of five categories – no myopic retinal degenerative lesion (category 0), tessellated fundus (category 1), diffuse chorioretinal atrophy (category 2), patchy chorioretinal atrophy (category 3), and macular atrophy (category 4). MMD-plus was defined as the presence of plus lesions – lacquer cracks, myopic choroidal neovascularization, and Fuchs spot [33].

### Measurement of choroidal thickness

Choroidal thickness measurements were obtained using Triton DRI-OCT (Topcon Medical Systems, Oakland, NJ, USA) and PLEX Elite 9000 (Carl Zeiss Meditec, Dublin, CA, USA). SS-OCT scans by both modalities were acquired within the same clinical visit to reduce unwanted effects of diurnal variation on choroidal thickness [34]. Notably, the Triton DRI-OCT uses a swept-source laser with an operational wavelength of 1050 nm and scanning speed of 100,000 A-scans per second. It has an axial and transverse resolution of 8 µm and 20 µm, respectively [35]. Similarly, the PLEX Elite 9000 utilizes a swept-source tuneable laser with an operational wavelength of 1040–1060 nm and scanning speed of 100,000 A-scans per second. It has a comparable axial and transverse resolution of 6.3 µm and 20 µm, respectively [36].

For Triton DRI-OCT, we employed the 3D Macula + Line (horizontal) scan protocol for image acquisition. The 3D macula imaging protocol scans an area of 7.0 × 7.0 mm with a resolution of 512 × 256, while the Line imaging protocol scans a length of 9.0 mm with a resolution of 1024 [35]. On the other hand, for PLEX Elite 9000, we employed the AngioPLEX™ protocol which scans an area of 3.0 × 3.0 mm [36]. For both OCT modalities, we performed manual and automated segmentation for choroidal thickness measurements.

For manual segmentation, we utilized the default lines plotted by the manufacturer’s proprietary software and subsequently manually adjusted them, if necessary. Segmentation lines were plotted at the Bruch’s membrane and choroidal–scleral interface. Measurement callipers were subsequently used to manually measure the distance between both segmentation lines subfoveally. To reduce inter-grader variability, choroidal thickness measurements were conducted independently by two trained graders. We masked the graders from the subject’s demographic data and ocular parameters to prevent intra-grader bias. Measurements from both graders were subsequently compared. If the inter-grader difference exceeded 10%, both graders collaboratively reviewed the image with a third adjudicator if necessary (Supplementary Fig. 1). Early Treatment Diabetic Retinopathy Study (ETDRS) grid choroidal thickness values derived from Triton’s proprietary software were also documented for Triton OCT images [37].

**Fig. 1:**
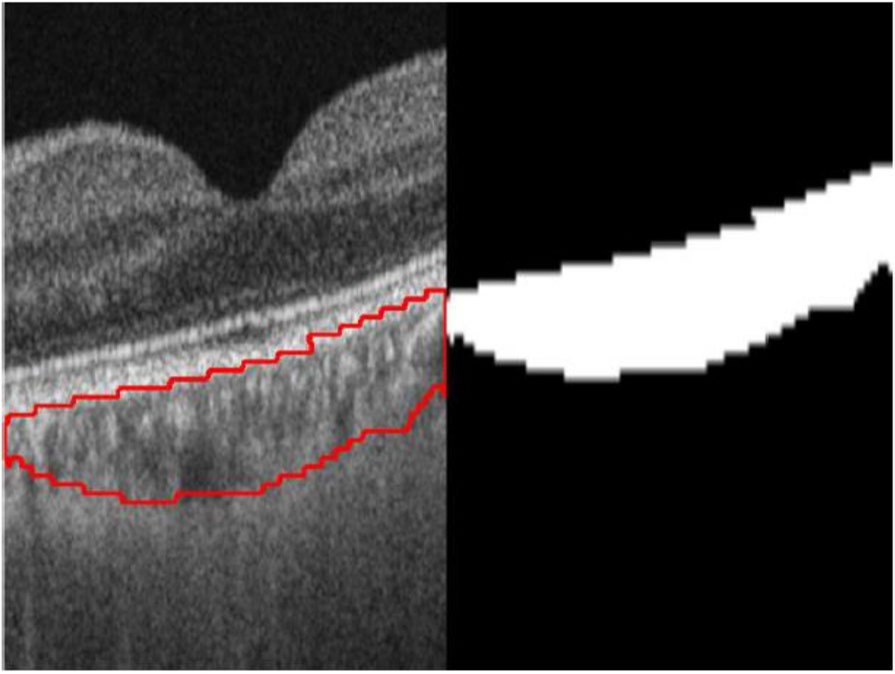
Automated segmentation by SA-Net

For automated segmentation, we utilized a previously described multi-task learning architecture – SA-Net (Fig. 1) [28]. In brief, this architecture comprises two branches, for reconstruction and segmentation. During reconstruction, the spatial context from adjacent cross-sectional slices are aggregated to form a central slice. Spatial context acquired is subsequently fused with a U-Net based architecture for segmentation. A five-fold cross-validation approach was adopted to train and assess the algorithm using a high myopia dataset [28]. Stratified sampling was performed over the choroidal volume for each fold to ensure that a similar distribution was achieved and to avoid dataset bias. To further avoid training bias and risk of overfitting, all images from the same eye were in the same fold. Further details can be found in the prior work [28]. Choroidal thickness measurements were then derived automatically based on the difference between the upper and lower bounds of the choroid. AI-automated segmentation was performed for both PLEX Elite 9000 and Triton DRI-OCT images, and each was compared to its own manual measurements.

### Statistical analysis

All statistical analyses were performed using SPSS statistical software (version 28; IBM, Chicago, IL, USA). Descriptive characteristics were calculated for those who met the inclusion criteria. Independent t-test and Chi-squared/ Fisher’s exact test were performed for continuous variables and categorical variables, respectively, to compare subject characteristics between different age groups (< 40 years versus ≥ 40 years) and axial length (< 26.0 mm versus ≥ 26.0 mm). Choroidal thickness (measured by Triton DRI-OCT) was further stratified into ETDRS grid areas. Multivariable linear regression analysis was also performed to evaluate the association between age, gender, and axial length with choroidal thickness across the different ETDRS grid areas. Linear mixed models were used to account for inter-eye correlation.

The Bland–Altman plot was used to illustrate the agreement between automated and manual segmented choroidal thickness measurements, in which the difference between choroidal thickness measurements (manual segmented measurements minus automated segmented measurements) was plotted against the mean value [38]. Proportion of outliers was determined by dividing the number of datapoints beyond the 95% limits of agreement (LOA) by the total number of measurements. Additionally, the one-sample t-test and linear regression model were performed to evaluate the presence of fixed and proportional bias, respectively. Intraclass correlation coefficient (ICC) was calculated based on a two-way mixed effects absolute agreement model to further assess the magnitude of agreement [39]. Pearson correlation coefficient was used to evaluate the correlation between automated and manual segmented choroidal thickness measurements. Correlation and agreement analyses were further stratified by axial length (< 26.0 mm versus ≥ 26.0 mm) and choroidal thickness (< 300 µm versus ≥ 300 µm). Similar analyses were performed to evaluate the agreement and correlation between choroidal thickness measurements from PLEX Elite 9000 and Triton DRI-OCT.

## Results

Two hundred and thirty-two subjects (464 eyes) underwent both Triton DRI-OCT and PLEX Elite 9000 scans. Eight eyes were excluded due to poor OCT scan quality. Consequently, 456 eyes from 229 subjects were included for analysis.

Table 1 details the demographics and ocular characteristics of the study subjects. The mean ± standard deviation (SD) age and visual acuity for subjects included for analysis were 34.1 ± 10.4 years and 0.02 ± 0.03 LogMAR, respectively. The mean ± SD subfoveal choroidal thickness based on manual segmentation was 216.9 ± 82.7 µm with Triton DRI-OCT and 239.3 ± 84.3 µm with PLEX Elite 9000. Those aged ≥ 40 years had significantly poorer visual acuity, less myopic spherical equivalent, thinner choroid, higher grade of PPA, higher grade of MMD, and higher prevalence of retinoschisis, posterior staphyloma, ERM, DSM, and intrachoroidal cavitation; they had a lower prevalence of peripheral retinal degeneration (all *P* ≤ 0.049). Subjects with axial length ≥ 26 mm were more likely to be male, had poorer visual acuity, higher myopic spherical equivalent, thinner choroid, higher grade of PPA, disc tilt, and MMD, as well as a higher prevalence of posterior staphyloma and DSM (all *P* ≤ 0.045).

**Table 1:** Comparison of baseline characteristics between subjects stratified by age and axial length

Demographics	Age < 40 years (*N* = 154)	Age ≥ 40 years (*N* = 75)	*P* value^†^	Axial length < 26 mm (*N* = 96)	Axial length ≥ 26 mm (*N* = 133)	*P* value^†^	Total (*N* = 229)
Age (years)	28.1 ± 6.2	46.5 ± 4.5	** < 0.001**	34.0 ± 10.5	34.2 ± 10.4	0.926	34.1 ± 10.4
Gender (male; n)	73 (47.4%)	28 (37.3%)	0.150	33 (34.4%)	68 (51.1%)	**0.012**	101 (44.1%)
Ethnicity (n)							
• Chinese	147 (95.5%)	73 (97.3%)	1.000	91 (94.8%)	129 (97.0%)	0.760	220 (96.1%)
• Malay	3 (1.9%)	1 (1.3%)		2 (2.1%)	2 (1.5%)		4 (1.7%)
• Indian	4 (2.6%)	1 (1.3%)		3 (3.1%)	2 (1.5%)		5 (2.2%)
**Ocular characteristics**	**Age < 40 years** **(** ***n*** **= 308)**	**Age ≥ 40 years** **(** ***n*** **= 148)**	***P*** ** value** ^†^	**Axial length < 26 mm** **(** ***n*** **= 188)**	**Axial length ≥ 26 mm** **(** ***n*** **= 268)**	***P*** ** value** ^†^	**Total** **(** ***n*** **= 456)**
**Ocular parameters**							
Visual acuity (LogMAR)	0.02 ± 0.02	0.03 ± 0.05	**0.021**	0.01 ± 0.02	0.03 ± 0.04	** < 0.001**	0.02 ± 0.03
Axial length (mm)	26.4 ± 1.4	26.4 ± 1.4	0.992	25.1 ± 0.6	27.3 ± 1.1	** < 0.001**	26.4 ± 1.4
Spherical equivalent (D)	− 6.2 ± 2.9	− 5.4 ± 3.8	**0.015**	− 4.4 ± 1.7	− 7.0 ± 3.6	** < 0.001**	− 5.9 ± 3.3
Choroidal thickness (μm)							
• Triton DRI-OCT	222.5 ± 79.9	205.3 ± 87.2	**0.038**	255.3 ± 82.0	190.1 ± 71.9	** < 0.001**	216.9 ± 82.7
• PLEX Elite 9000	245.9 ± 82.5	225.6 ± 86.5	**0.016**	278.7 ± 83.6	211.6 ± 73.1	** < 0.001**	239.3 ± 84.3
**Optic disc**							
Peripapillary atrophy (n)							
• None	21 (6.8%)	7 (4.7%)	**0.012**	21 (11.2%)	7 (2.6%)	** < 0.001**	28 (6.1%)
• Mild	176 (57.1%)	74 (50.0%)		117 (62.2%)	133 (49.6%)		250 (54.8%)
• Moderate	110 (35.7%)	61 (41.2%)		50 (26.6%)	121 (45.1%)		171 (37.5%)
• Severe	1 (0.3%)	6 (4.1%)		0	7 (2.6%)		7 (1.5%)
Disc tilt (n)							
• None	35 (11.4%)	11 (7.4%)	0.419	34 (18.1%)	12 (4.5%)	** < 0.001**	46 (10.1%)
• Mild	237 (76.9%)	117 (79.1%)		142 (75.5%)	212 (79.1%)		354 (77.6%)
• Moderate	34 (11.0%)	20 (13.5%)		12 (6.4%)	42 (15.7%)		54 (11.8%)
• Severe	2 (0.6%)	0		0	2 (0.7%)		2 (0.4%)
**Macula**							
Myopic macular degeneration (n)							
• None	73 (23.7%)	23 (15.5%)	**0.049**	68 (36.2%)	28 (10.4%)	** < 0.001**	96 (21.1%)
• Tessellated Fundus	218 (70.8%)	111 (75.0%)		118 (62.8%)	211 (78.7%)		329 (72.1%)
• Diffuse peripapillary/ chorioretinal atrophy	17 (5.5%)	13 (8.8%)		2 (1.1%)	28 (10.4%)		30 (6.6%)
• Patchy chorioretinal atrophy	0	1 (0.7%)		0	1 (0.4%)		1 (0.2%)
• Macular atrophy	0	0		0	0		0
Plus lesions (n)	0	1 (0.7%)	0.325	0	1 (0.4%)	1.000	1 (0.2%)
Macular hole (n)	0	1 (0.7%)	0.325	0	1 (0.4%)	1.000	1 (0.2%)
Myopic tractional maculopathy (n)	0	0	-	0	0	-	0
**Retina**							
Peripheral retina degeneration (n)							
• None	285 (92.5%)	148 (100.0%)	**0.002**	180 (95.7%)	253 (94.4%)	0.427	433 (95.0%)
• White without pressure	12 (3.9%)	0		3 (1.6%)	9 (3.4%)		12 (2.6%)
• Lattice degeneration	10 (3.2%)	0		4 (2.1%)	6 (2.2%)		10 (2.2%)
• Retinal holes and tears	1 (0.3%)	0		1 (0.5%)	0		1 (0.2%)
• Retinal detachment	0	0		0	0		0
Retinoschisis (n)	0	3 (2.0%)	**0.034**	0	3 (1.1%)	0.271	3 (0.7%)
Posterior staphyloma (n)	4 (1.3%)	8 (5.4%)	**0.023**	0	12 (4.5%)	**0.002**	12 (2.6%)
Epiretinal membrane (n)	2 (0.6%)	6 (4.1%)	**0.016**	2 (1.1%)	6 (2.2%)	0.480	8 (1.8%)
Dome shaped macula (n)	0	7 (4.7%)	** < 0.001**	0	7 (2.6%)	**0.045**	7 (1.5%)
Intrachoroidal cavitation (n)	0	3 (2.0%)	**0.034**	2 (1.1%)	1 (0.4%)	0.572	3 (0.7%)

Data presented as mean ± standard deviation or number (percentage), where appropriate

*N* = number of subjects; *n* = number of eyes

*P* values in bold indicate statistical significance

^†^ *P* value was estimated based on Chi-squared, Fisher’s exact, or independent t-test, where appropriate

**Fig. 2:**
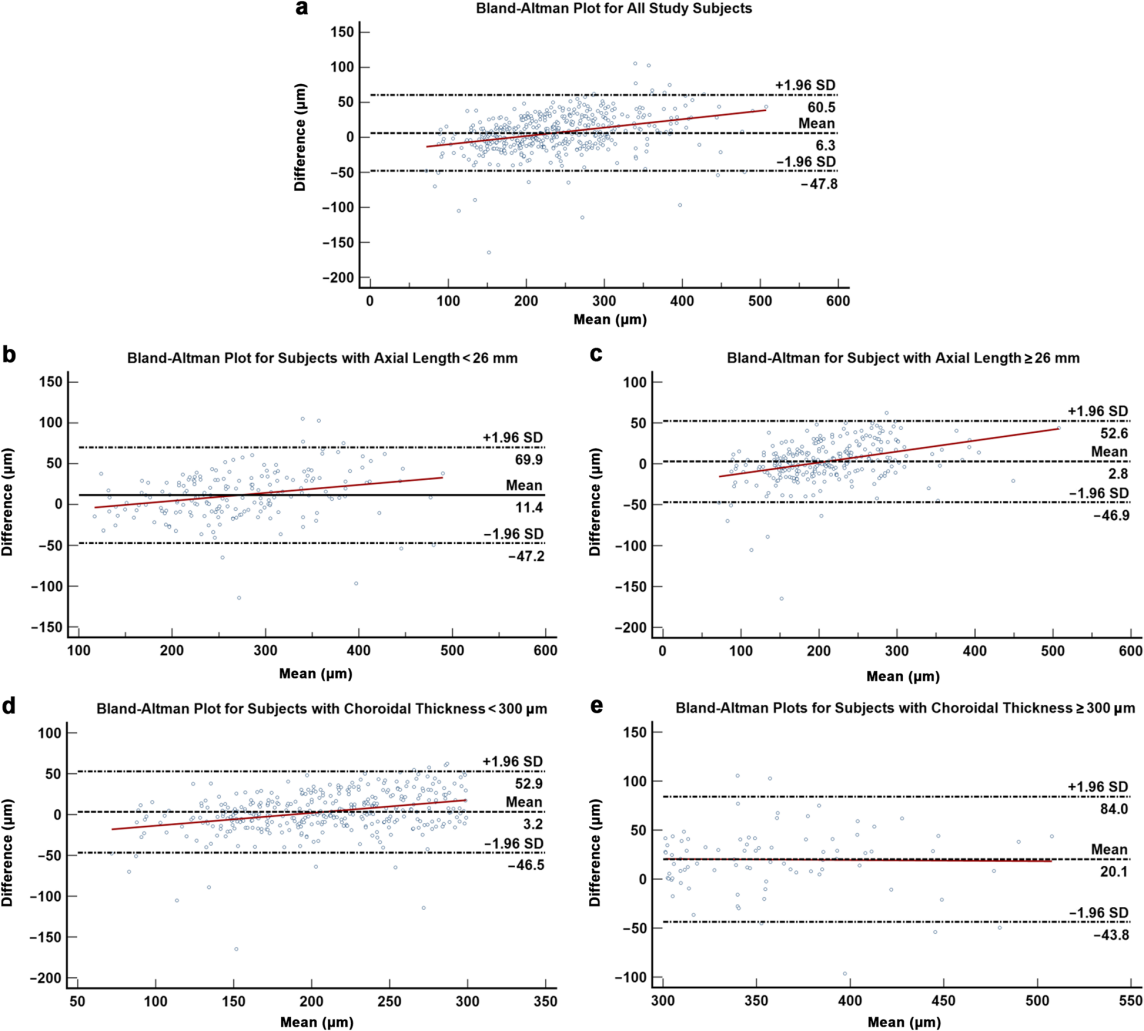
Bland–Altman plot demonstrating the agreement between automated and manual segmented choroidal thickness measurements for PLEX Elite 9000. **a** All subjects; **b**, **c** Subjects with axial length less than and more than 26 mm, respectively; **d**, **e** Subjects with choroidal thickness less than and more than 300 μm, respectively

**Fig. 3:**
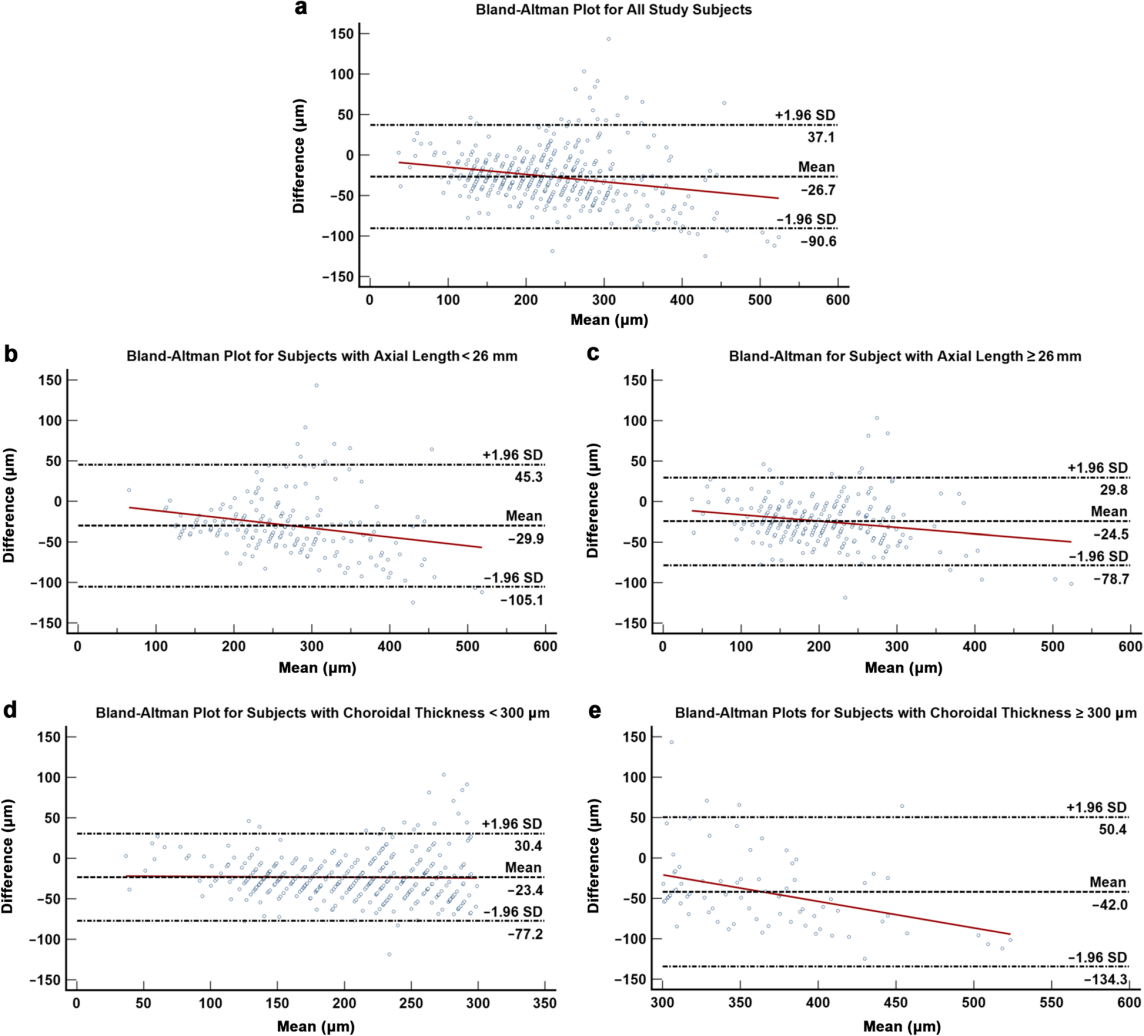
Bland–Altman plot demonstrating the agreement between automated and manual segmented choroidal thickness measurements for DRI-OCT. **a** All subjects; **b**, **c** Subjects with axial length less than and more than 26 mm, respectively; **d**,** e** Subjects with choroidal thickness less than and more than 300 μm, respectively

Table 2 details the agreement and correlation between manual and automated segmented choroidal thickness measurements for Triton DRI-OCT and PLEX Elite 9000. Both modalities demonstrated excellent agreement (PLEX Elite 9000: ICC = 0.937, 95% CI: 0.922 to 0.949, *P* < 0.001; Triton DRI-OCT: ICC = 0.887, 95% CI: 0.608 to 0.950, *P* < 0.001) and correlation (PLEX Elite 9000: *r* = 0.946, *P* < 0.001; Triton DRI-OCT: *r* = 0.933, *P* < 0.001) between manual and automated segmented choroidal thickness measurements. For PLEX Elite 9000, manual segmented measurements were generally thicker as compared with automated segmented measurements, with a fixed bias of 6.344 µm (95% CI: 3.802 to 8.886, *P* < 0.001, 95% LOA =  − 47.787 to 60.475; Fig. 2a) and proportional bias of 0.120 (*P* < 0.001). On the contrary, Triton DRI-OCT manual segmented measurements were comparatively thinner than automated segmented measurements, with a fixed bias of − 26.714 µm (95% CI: − 29.711 to − 23.716, *P* < 0.001, 95% LOA =  − 90.556 to 37.127; Fig. 3a) and proportional bias of − 0.090 (*P* < 0.001). Agreement and correlation between manual and automated segmented choroidal thickness remained excellent following stratification for axial length (< 26 mm versus ≥ 26 mm; Figs. 2b, 2c, 3b, and 3c). On the other hand, following stratification for choroidal thickness, subjects with choroidal thickness ≥ 300 µm demonstrated weaker agreement and correlation with a larger magnitude of fixed bias when compared with those with choroidal thickness < 300 µm (Figs. 2d, 2e, 3d, and 3e).

**Table 2:** Agreement and correlation between automated and manual segmented choroidal thickness

**Subjects**	**n**	**LOA**		**Proportion of outliers (n [%])**	**Fixed bias**	**95% CI**	***P*** ** value**	**Proportional bias (β)**	***P*** ** value**	**ICC**^a^	**95% CI**	***P*** ** value**	**r**	***P*** ** value**
		**Lower bound LOA** **(95% CI)**	**Upper bound LOA** **(95% CI)**											
**PLEX Elite 9000**														
All subjects	456	− 47.787 (− 52.134 to − 43.440)	60.475 (56.128 to 64.822)	20 (4.4)	6.344	3.802 to 8.886	** < 0.001**	0.120	** < 0.001**	0.937	0.922 to 0.949	** < 0.001**	0.946	** < 0.001**
AL < 26 mm	188	− 47.171 (− 54.530 to − 39.813)	69.932 (62.574 to 77.291)	9 (4.8)	11.380	7.083 to 15.679	** < 0.001**	0.098	** < 0.001**	0.921	0.878 to 0.947	** < 0.001**	0.934	** < 0.001**
AL ≥ 26 mm	268	− 46.943 (− 52.168 to − 41.719)	52.565 (47.341 to 57.789)	8 (3.0)	2.811	− 0.242 to 5.864	0.071	0.134	** < 0.001**	0.931	0.913 to 0.946	** < 0.001**	0.940	** < 0.001**
ChT < 300 µm	371	− 46.524 (− 50.955 to − 42.940)	52.923 (48.493 to 57.353)	13 (3.5)	3.199	0.610 to 5.789	**0.016**	0.158	** < 0.001**	0.890	0.866 to 0.909	** < 0.001**	0.901	** < 0.001**
ChT ≥ 300 µm	85	− 43.815 (− 55.877 to − 31.753)	83.952 (71.890 to 96.014)	6 (7.1)	20.068	13.038 to 27.099	** < 0.001**	− 0.011	0.881	0.749	0.492 to 0.864	** < 0.001**	0.803	** < 0.001**
**Triton DRI-OCT**														
All subjects	456	− 90.556 (− 95.683 to − 85.429)	37.127 (32.000 to 42.254)	30 (6.6)	− 26.714	− 29.711 to − 23.716	** < 0.001**	− 0.090	** < 0.001**	0.887	0.608 to 0.950	** < 0.001**	0.933	** < 0.001**
AL < 26 mm	188	− 105.076 (− 114.522 to − 95.629)	45.258 (35.811 to 54.704)	11 (5.9)	− 29.908	− 35.426 to − 24.391	** < 0.001**	− 0.108	**0.001**	0.852	0.555 to 0.931	** < 0.001**	0.907	** < 0.001**
AL ≥ 26 mm	268	− 78.709 (− 84.403 to − 73.014)	29.762 (24.067 to 35.456)	14 (5.2)	− 24.473	− 27.801 to − 21.145	** < 0.001**	− 0.077	** < 0.001**	0.884	0.545 to 0952	** < 0.001**	0.934	** < 0.001**
ChT < 300 µm	375	− 77.249 (− 82.020 to − 72.479)	30.419 (25.648 to 35.189)	17 (4.5)	− 23.415	− 26.204 to − 20.626	** < 0.001**	− 0.011	0.666	0.830	0.453 to 0.924	** < 0.001**	0.894	** < 0.001**
ChT ≥ 300 µm	81	− 134.339 (− 152.218 to − 116.461)	50.362 (32.484 to 68.241)	6 (7.4)	− 41.987	− 52.406 to − 31.569	** < 0.001**	− 0.328	** < 0.001**	0.561	0.090 to 0.778	** < 0.001**	0.723	** < 0.001**

*P* values in bold indicate statistical significance

*n* = number of eyes; *LOA* = limits of agreement; *β* = unstandardized coefficient; *r* = Pearson correlation coefficient; *ICC* = intraclass correlation coefficient; *AL* = axial length; *ChT* = choroidal thickness

^a^ICC was calculated based on two-way mixed effects absolute agreement model

**Fig. 4:**
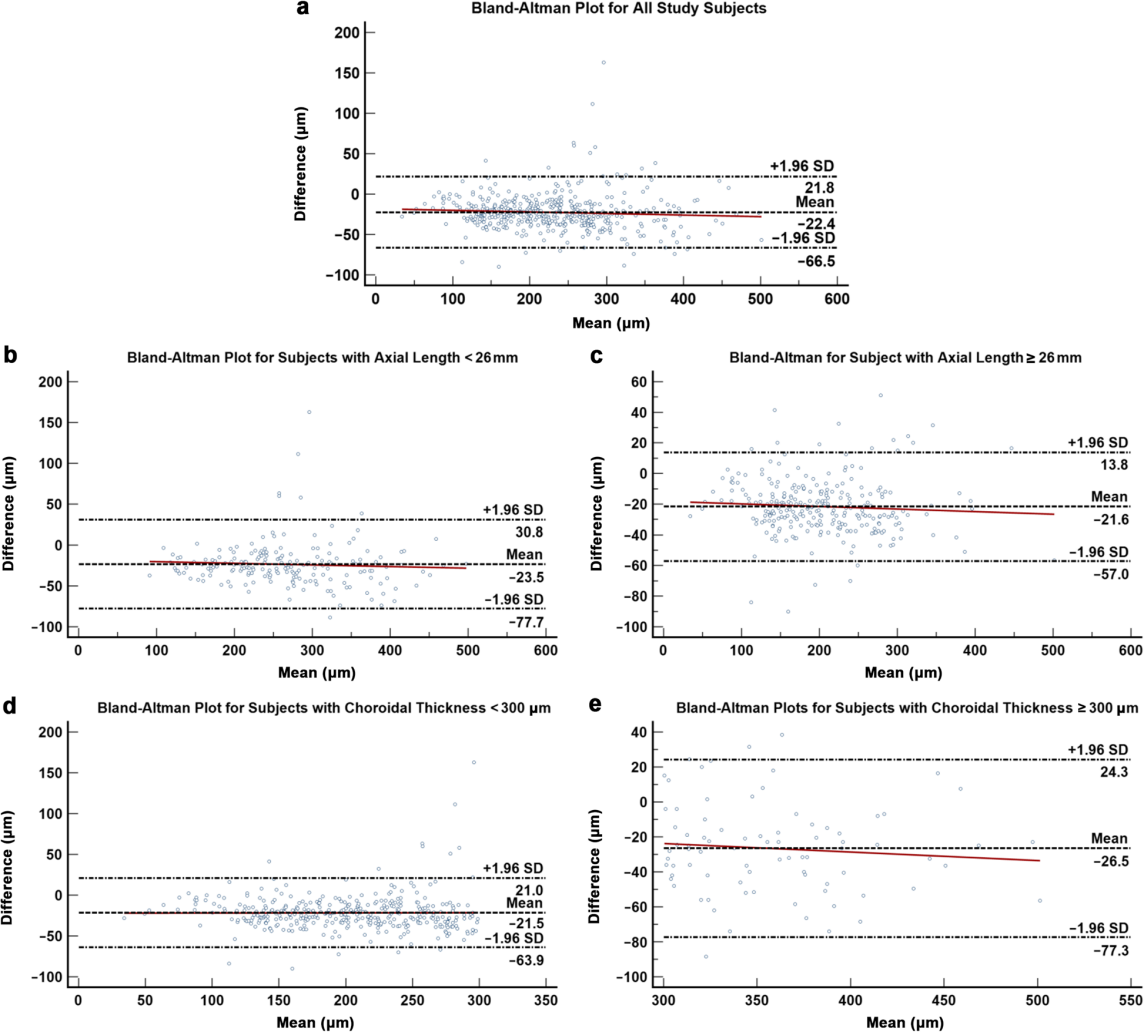
Bland–Altman plot demonstrating the agreement between Triton DRI-OCT and PLEX Elite 9000 for choroidal thickness measurements. **a** all subjects; **b**, **c** Subjects with axial length less than and more than 26 mm, respectively; **d**, **e** Subjects with choroidal thickness less than and more than 300 μm, respectively

Table 3 summarizes the indices for agreement and correlation between Triton DRI-OCT and PLEX Elite 9000 for manual-segmented choroidal thickness measurement. ICC (0.930, 95% CI: 0.602 to 0.974, *P* < 0.001) and Pearson correlation coefficient (*r* = 0.964, *P* < 0.001) demonstrated excellent agreement and correlation between both SS-OCT modalities. Triton DRI-OCT choroidal thickness measurements were comparatively thinner than PLEX Elite 9000, with a fixed bias of − 22.363 µm (95% CI: − 24.435 to − 20.291, *P* < 0.001, 95% LOA: − 66.501 to 21.775; Fig. 4a). Nevertheless, there was no statistically significant proportional bias (*P* = 0.126) between the two SS-OCT modalities. Following stratification for axial length (< 26 mm versus ≥ 26 mm), the inter-modality agreement and correlation coefficient remained excellent in both subgroups. Subjects with axial length < 26 mm (fixed bias =  − 23.457, 95% CI: − 27.439 to − 19.476, *P* < 0.001, 95% LOA: − 77.698 to 30.783) showed a larger magnitude of fixed bias as compared to subjects with axial length ≥ 26 mm (fixed bias =  − 21.595, 95% CI: − 23.768 to − 19.422, *P* < 0.001, 95% LOA: − 57.005 to 13.815; Fig. 4b and c). No proportional bias was observed in either subgroup (all *P* ≥ 0.273). On the contrary, when the analysis was stratified for choroidal thickness, subjects with choroidal thickness ≥ 300 µm (ICC = 0.759, 95% CI: 0.175 to 0.905, *P* < 0.001; *r* = 0.866,* P* < 0.001) demonstrated weaker correlation and agreement as compared to those with choroidal thickness < 300 µm (ICC = 0.874, 95% CI: 0.423 to 0.951, *P* < 0.001; *r* = 0.932, *P* < 0.001). Notably, the magnitude of fixed bias was larger in subjects with choroidal thickness ≥ 300 µm (fixed bias =  − 26.512, 95% CI: − 32.241 to − 20.783, *P* < 0.001, 95% LOA: − 77.295 to 24.270 versus fixed bias =  − 21.467, 95% CI: − 23.665 to − 19.268, *P* < 0.001, 95% LOA: − 63.906 to 20.972; Fig. 4d and e). Proportional bias was statistically insignificant in both subgroups (all *P* ≥ 0.421).

**Table 3:** Agreement and correlation between triton DRI-OCT and PLEX Elite 9000 for manual-segmented choroidal thickness measurement

**Subjects**	**n**	**LOA**		**Proportion of outliers (n [%])**	**Fixed bias**	**95% CI**	***P*** ** value**	**Proportional bias (β)**	***P*** ** value**	**ICC** ^a^	**95% CI**	***P*** ** value**	**r**	***P*** ** value**
		**Lower bound LOA** **(95% CI)**	**Upper bound LOA** **(95% CI)**											
All subjects	456	− 66.501 (− 70.045 to − 62.957)	21.775 (18.231 to 25.320)	23 (5.0)	− 22.363	− 24.435 to − 20.291	** < 0.001**	− 0.020	0.126	0.930	0.602 to 0.974	** < 0.001**	0.964	** < 0.001**
AL < 26 mm	188	− 77.698 (− 84.515 to − 70.882)	30.783 (23.967 to 37.600)	7 (3.7)	− 23.457	− 27.439 to − 19.476	** < 0.001**	− 0.019	0.442	0.908	0.639 to 0.961	** < 0.001**	0.944	** < 0.001**
AL ≥ 26 mm	268	− 57.005 (− 60.723 to − 53.287)	13.815 (10.097 to 17.533)	18 (6.7)	− 21.595	− 23.768 to − 19.422	** < 0.001**	− 0.017	0.273	0.928	0.416 to 0.976	** < 0.001**	0.969	** < 0.001**
ChT < 300 µm	375	− 63.906 (− 67.666 to − 60.145)	20.972 (17.211 to 24.733)	14 (3.7)	− 21.467	− 23.665 to − 19.268	** < 0.001**	0.001	0.946	0.874	0.423 to 0.951	** < 0.001**	0.932	** < 0.001**
ChT ≥ 300 µm	81	− 77.295 (− 87.126 to − 67.464)	24.270 (14.439 to 34.102)	4 (4.9)	− 26.512	− 32.241 to − 20.783	** < 0.001**	− 0.049	0.421	0.759	0.175 to 0.905	** < 0.001**	0.866	** < 0.001**

*P* values in bold indicate statistical significance

*n* = number of eyes; *LOA* = limits of agreement; β = unstandardized coefficient; *r* = Pearson correlation coefficient; *ICC* = intraclass correlation coefficient; *AL* = axial length; *ChT* = choroidal thickness

^a^ICC was calculated based on two-way mixed effects, absolute agreement model

## Discussion

In our study of myopic adults, we observed a mean choroidal thickness of 216.9 µm and 239.3 µm measured by the Triton DRI-OCT and PLEX Elite 9000, respectively. Both modalities exhibited excellent agreement between automated (SA-Net) and manual segmented choroidal thickness measurements in myopic adults with a range of different axial lengths. Notably, the magnitude of agreement was markedly lower in eyes with thicker choroids (≥ 300 µm). Choroidal thickness measurements by both SS-OCT modalities were also comparable with excellent agreement indices. To the best of our knowledge, our study is the first to demonstrate the agreement between these measurements. Our findings contribute further insights into the normative choroidal thickness amongst a population of myopes and provide further clarification towards the clinical utility of SA-Net and interchangeability of choroidal thickness measurements across different SS-OCT modalities.

Several studies have described the normative profiles of choroidal thickness in myopic eyes [7, 40–49]. Given the heterogenous subject characteristics and study methodologies (OCT modality and myopia definition), it is challenging to compare our mean choroidal thickness values with other studies. However, we were able to replicate the previously demonstrated relationships between both increased age and axial length with thinner choroidal thickness [7, 40–49]. When we further stratified the analysis based on ETDRS grid areas, we observed that choroidal thickness was thinnest in the nasal macular region (Supplementary Table 1), consistent with several other studies [47–54]. This may be explained by the choroid’s watershed zone, which is located between the optic disc and the fovea [55, 56]. Interestingly, following adjustment for age and gender, we also observed that the change in choroidal thickness per unit change in axial length was the greatest in the inferior macular regions (Supplementary Table 2; β =  − 29.188 and β =  − 28.256 in the inner and outer macular areas, respectively), suggesting that these areas could be more susceptible to stretching and subsequent thinning with axial length elongation. However, due to the cross-sectional nature of our study, further longitudinal studies are necessary to validate these hypotheses.

AI has recently garnered significant interest in ophthalmology, driving the automation of several clinical processes [57–65]. In this regard, several deep-learning based approaches have been proposed for the segmentation of 3D volumetric data such as OCT images [28–31]. Nonetheless, the majority of these methods require extensive computational memory and long processing time, making them less feasible to adopt in the clinical setting. Cahyo et al. have introduced a novel multi-task learning approach – SA-Net, targeted towards addressing these limitations [28]. We compared our manual segmented choroidal thickness measurements to those segmented by SA-Net [28] and observed an excellent agreement, albeit with an exception in subjects with choroidal thickness ≥ 300 µm. Importantly, patients with thinner choroids are more at risk of pathology, and thus our results hold well for the targeted patient population, which would be followed more closely in a clinical setting.

As proposed by Tan et al., a thicker choroid may result in higher signal loss and more artefacts owing to a larger amount of interstitial connective tissue [20]. In this regard, visualization of the choroidal–scleral interface may be impacted, thereby leading to greater variability during manual segmentation [24]. On the contrary, Cahyo et al. evaluated the accuracy of segmentation volume and resemblance of thickness map with respect to ground truth segmentation and observed better segmentation performance in thicker choroids (≥ 300 µm) across all AI-driven architectures [28]. Collectively, this may suggest that the visibility of Bruch’s membrane and choroidal–scleral interface perceived by the human eye and AI may depend on different factors and choroidal thickness measurements in subjects with thicker choroid may be more accurate with SA-Net.

Apart from being able to perform automated choroidal segmentation with comparable accuracy to manual segmentation, Cahyo et al.’s novel algorithm also possesses the intrinsic advantage accompanied with AI – superior repeatability, reduced grader variability, and less labour intensiveness. Considering its strengths, automated segmentation may become the mainstay method for determining choroidal thickness clinically. Nonetheless, future studies are necessary to further evaluate this aspect.

Our secondary aim was to evaluate the correlation and agreement between two well-established SS-OCT modalities – Triton DRI-OCT and PLEX Elite 9000. In terms of correlation, our findings (*r* = 0.964, *P* < 0.001) were consistent with Marenco et al.’s study, which also found an excellent correlation between choroidal thickness measurements from both modalities (*r* = 0.944, *P* < 0.001), albeit in a study population of subjects with primary open-angle glaucoma [66]. Although the agreement between both modalities was determined to be excellent in our study, clinicians should be cognizant of the inter-modality fixed bias (− 22.363 µm), particularly in selected clinical context. For instance, in OCT-based staging of MMD, choroidal thickness for peripapillary and macular diffuse choroidal atrophy was 84.6 μm and 50.2 μm, respectively, and thus a magnitude of − 22.363 µm may well be significant in evaluating pathologic myopes [15].

We postulate that this fixed bias may be attributed to the differences in the axial resolution of these modalities – 8 µm in Triton DRI-OCT versus 6.3 µm in PLEX Elite 9000 [35, 36]. The axial resolution, as defined by the ability to distinguish between two distinct objects which are positioned adjacent to each other in the longitudinal plane, may influence the visualization of the true choroidal–scleral interface. In this regard, measurements derived from the PLEX Elite 9000 may potentially be a closer representation of the true choroidal thickness. Nevertheless, further studies are warranted to ascertain this finding.

Based on previous OCT studies, axial length was found to have an impact on the optical magnification and accuracy of OCT-derived ocular parameters, namely due to transverse magnification from axial length [67, 68]. Hence, we stratified our analysis to elucidate the effect of axial length on the agreement between two modalities. We found minimal differences in Pearson correlation coefficient and ICC, suggesting that both modalities may be equally affected in this aspect. We also stratified our analysis for choroidal thickness and observed weaker correlation and agreement indices in subjects with thicker choroids (≥ 300 µm), albeit still high with a coefficient of 0.866 and 0.759, respectively. Furthermore, subjects with thicker choroids also showed a larger magnitude of fixed bias, suggesting more variability within measurements. As discussed earlier in this section, this may be due to the higher signal loss and increased artefacts associated with thicker choroids [20, 24]. Taken together, inter-modality choroidal thickness measurements must be interpreted with care in subjects with thicker choroids.

The strengths of our study include its large sample size of myopic subjects with a broad range of axial length and spherical equivalent. Furthermore, a robust study design with standardized methodology was adopted, with conscious efforts to reduce intra- and inter-grader variability. To our knowledge, this is the first study comparing choroidal thickness measurements in these two commonly used imaging modalities. Nevertheless, our study has its limitations. Our study lacks representation from older subjects, who might be the more important target population given their higher risk of thin choroid and pathologic myopia. Moreover, our study population is generally healthy with an average visual acuity of 0.02 LogMAR, so these results may not be generalizable to those with ocular disease such as pathologic myopia. The exclusion of images of poor quality from our study might also limit the generalizability of our findings to real-world clinical scenarios, where encountering poor-quality images is inevitable, particularly in high myopes with anatomical variances such as staphyloma, DSM, and severe choroidal thinning. Notably, both SS-OCT modalities adopted significantly different imaging protocol. Given that the imaging protocols are proprietary to respective manufacturers, this is an inherent limitation within our study. Nonetheless, this mirrors a real-world scenario encountered in the clinical settings, where different modalities often employ distinct imaging protocols. In this regard, SA-Net only analyses the foveal and macular area (Fig. 1), meaning the difference in scan dimensions between Triton DRI-OCT and PLEX Elite 9000 (3.0 × 3.0 mm versus 7.0 × 7.0 mm) is unlikely to impact the comparison between modalities. We also did not directly correct for transverse magnification due to axial length, although both modalities would be affected by this bias. Furthermore, due to the lack of topographical choroidal thickness data from the PLEX Elite 9000, we were only able to ascertain the agreement and correlation between Triton DRI-OCT and PLEX Elite 9000 for subfoveal choroidal thickness. Further studies examining choroidal thickness data across the macular region are warranted to validate this aspect. It is essential to acknowledge that SA-Net assumed the foveal location to be precisely at the center of the images. Although most images were generally well-centered in our study, this could potentially introduce minor offsets, which could have affected the accuracy of SFCT measurements. Future studies may utilise adjunctive registration software to improve fovea detection and accuracy of SFCT measurements. Our study would have also provided more insight by including time as an objective measure, allowing for an evaluation of the differences in duration required for manual versus automated segmentation. Lastly, as manual segmented choroidal thickness measurements were performed with callipers, issues with repeatability cannot be excluded.

## Conclusion

Choroidal thickness measurements using manual and automated (SA-Net) segmentation are comparable among adults with myopia. Given its edge over manual segmentation, automated segmentation may further enhance the clinical utility of choroidal thickness in myopia management and emerge as the primary method of measurement in the future.

### Supplementary Information

**Additional file 1:** **Supplementary Figure 1.** Protocol for the manual measurement of choroidal thickness with Triton DRI-OCT and PLEX Elite 9000.

**Additional file 2:** **Supplementary Table 1.** Choroidal thickness measured with Triton DRI-OCT, across Early Treatment Diabetic Retinopathy Study (ETDRS) grid areas.

**Additional file 3:** **Supplementary Table 2.** Multivariable linear regression with linear mixed model analysis on the associations between age, gender, and axial length with choroidal thickness across the ETDRS grid areas.

## Data Availability

The datasets used and/or analysed in this current study are available from the corresponding author on reasonable request.
