# Supplementary material for: Comparison of manual and artificial intelligence-automated choroidal thickness segmentation of optical coherence tomography imaging in myopic adults
Source: Eye Vis (Lond). 2024 Jun 3;11:21. doi: 10.1186/s40662-024-00385-2 (PMC11145894; doi:10.1186/s40662-024-00385-2)
Supplement: Supplementary file 1 — Additional file 1: Supplementary Figure 1. Protocol for the manual measurement of choroidal thickness with Triton DRI-OCT and PLEX Elite 9000. [file 40662_2024_385_MOESM1_ESM.pdf]

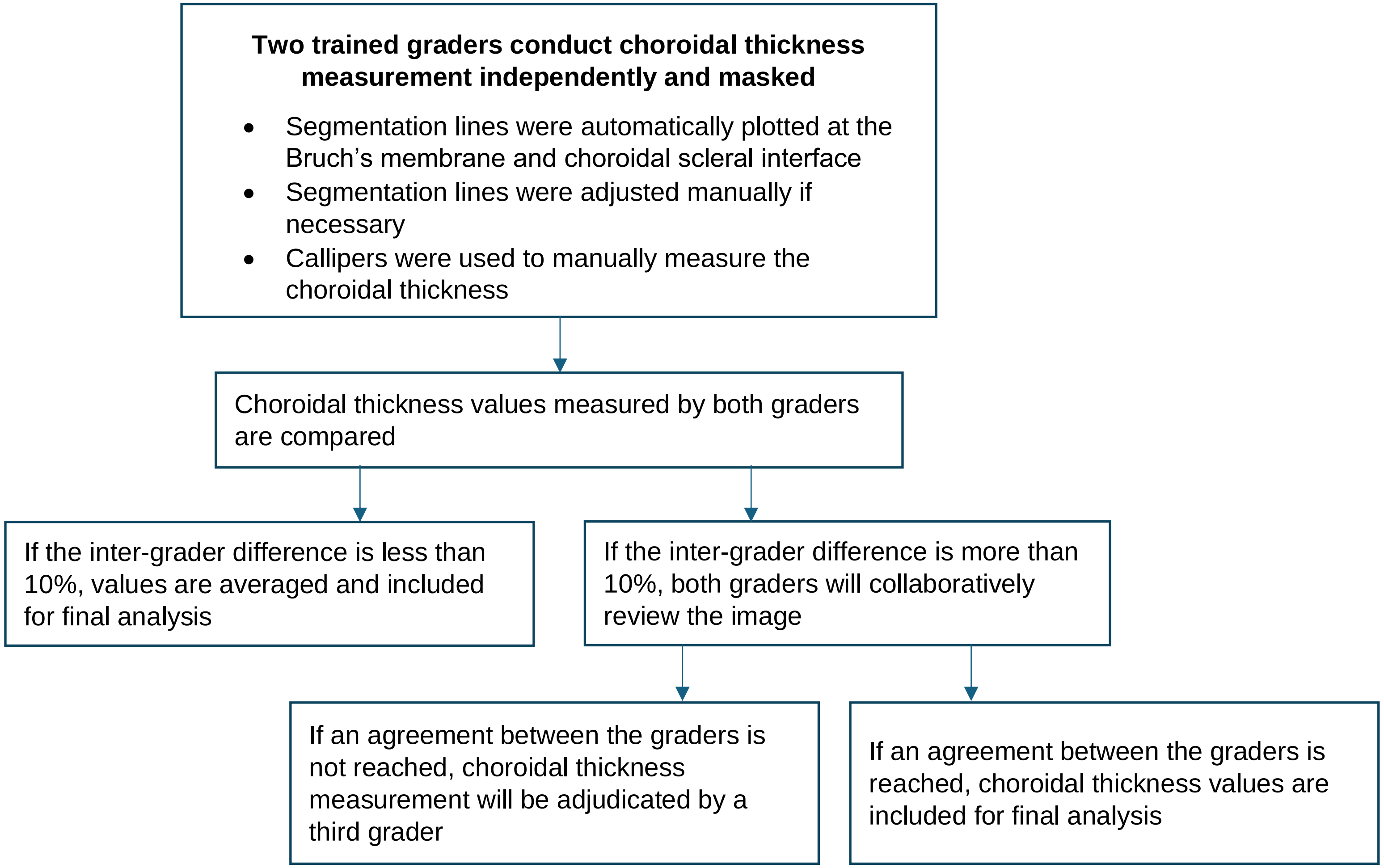
Two trained graders conduct choroidal thickness measurement independently and masked
Segmentation lines were automatically plotted at the Bruch’s membrane and choroidal scleral interface
Segmentation lines were adjusted manually if necessary
Callipers were used to manually measure the choroidal thickness
Choroidal thickness values measured by both graders are compared
If the inter-grader difference is less than 10%, values are averaged and included for final analysis
If the inter-grader difference is more than 10%, both graders will collaboratively review the image
If an agreement between the graders is not reached, choroidal thickness measurement will be adjudicated by a third grader
If an agreement between the graders is reached, choroidal thickness values are included for final analysis
