# Supplementary material for: Comparison of manual and artificial intelligence-automated choroidal thickness segmentation of optical coherence tomography imaging in myopic adults
Source: Eye Vis (Lond). 2024 Jun 3;11:21. doi: 10.1186/s40662-024-00385-2 (PMC11145894; doi:10.1186/s40662-024-00385-2)
Supplement: Supplementary file 2 — Additional file 2: Supplementary Table 1. Choroidal thickness measured with Triton DRI-OCT, across Early Treatment Diabetic Retinopathy Study (ETDRS) grid areas. [file 40662_2024_385_MOESM2_ESM.docx]

**Supplementary Table 1. Choroidal thickness measured with Triton DRI-OCT, across Early Treatment Diabetic Retinopathy Study (ETDRS) grid areas**

|  | **Choroidal thickness (μm)** | | | | | | |
| --- | --- | --- | --- | --- | --- | --- | --- |
| **ETDRS grid areas** | **Age below 40 years**  **(n=308)** | **Age above 40 years**  **(n=148)** | ***P* value†** | **Axial length**  **< 26 mm**  **(n=188)** | **Axial length**  **≥ 26 mm**  **(n=268)** | ***P* value†** | **Total** |
| **Central** | 209.9±73.1 | 192.3±80.1 | **0.025** | 237.4±76.5 | 180.9±66.2 | **<0.001** | 204.2±75.8 |
| **Inner macular ring** |  |  |  |  |  |  |  |
| - Superior | 220.8±74.4 | 199.4±77.3 | **0.005** | 241.4±76.3 | 194.5±69.7 | **<0.001** | 213.8±75.9 |
| - Inferior | 208.1±74.2 | 189.7±81.4 | **0.017** | 235.9±77.1 | 178.5±67.7 | **<0.001** | 202.2±77.0 |
| - Temporal | 224.3±71.4 | 201.1±78.1 | **0.002** | 247.7±74.3 | 195.1±66.3 | **<0.001** | 216.8±74.3 |
| - Nasal | 186.9±70.5 | 174.1±76.1 | 0.078 | 216.4±72.2 | 159.2±62.9 | **<0.001** | 182.7±72.5 |
| **Outer macular ring** |  |  |  |  |  |  |  |
| - Superior | 233.5 ±74.6 | 211.8±74.8 | **0.004** | 248.9±73.1 | 210.7±72.8 | **<0.001** | 226.5±75.3 |
| - Inferior | 206.4±72.2 | 183.5±73.4 | **0.002** | 230.8±73.2 | 176.7±64.7 | **<0.001** | 199.0±73.3 |
| - Temporal | 229.6±65.9 | 205.1±69.1 | **<0.001** | 247.7±68.1 | 203.4±61.6 | **<0.001** | 221.6±67.9 |
| - Nasal | 146.8±61.7 | 137.5±67.0 | 0.147 | 173.2±64.5 | 123.1±54.1 | **<0.001** | 143.8±63.5 |

ETDRS = Early Treatment Diabetic Retinopathy Study; n = number of eyes.
Data presented as mean ± standard deviation.

†*P* value was estimated based on independent t-test.

*P* values in bold indicate statistical significance.
