# Supplementary material for: Comparison of manual and artificial intelligence-automated choroidal thickness segmentation of optical coherence tomography imaging in myopic adults
Source: Eye Vis (Lond). 2024 Jun 3;11:21. doi: 10.1186/s40662-024-00385-2 (PMC11145894; doi:10.1186/s40662-024-00385-2)
Supplement: Supplementary file 3 — Additional file 3: Supplementary Table 2. Multivariable linear regression with linear mixed model analysis on the associations between age, gender, and axial length with choroidal thickness across the ETDRS grid areas. [file 40662_2024_385_MOESM3_ESM.docx]

**Supplementary Table 2. Multivariable linear regression with linear mixed model analysis on the associations between age, gender, and axial length with choroidal thickness across the ETDRS grid areas**

| **n=456** | **Central** | | | **Inner macula** | | | | | | | | | | | |
| --- | --- | --- | --- | --- | --- | --- | --- | --- | --- | --- | --- | --- | --- | --- | --- |
|  |  |  |  | **Superior** | | | **Inferior** | | | **Temporal** | | | **Nasal** | | |
| **Predictor variables** | **β** | **95% CI** | ***P* value** | **β** | **95% CI** | ***P***  **value** | **β** | **95% CI** | ***P***  **value** | **β** | **95% CI** | ***P***  **value** | **β** | **95% CI** | ***P***  **value** |
| Age | −0.851 | −1.622 to −0.080 | **0.031** | −1.069 | −1.869 to −0.268 | **0.009** | −0.966 | −1.734 to −0.199 | **0.014** | −1.238 | −1.982 to −0.493 | **0.001** | −0.569 | −1.330 to 0.191 | 0.142 |
| Gender | 29.943 | 13.508 to 46.379 | **<0.001** | 30.649 | 13.593 to 47.704 | **<0.001** | 29.409 | 13.051 to 45.767 | **<0.001** | 30.110 | 14.243 to 45.976 | **<0.001** | 22.939 | 6.753 to 39.124 | **0.006** |
| Axial length | −27.778 | −33.131 to −22.426 | **<0.001** | −26.323 | −31.822 to −20.825 | **<0.001** | −29.188 | −34.524 to −23.852 | **<0.001** | −27.543 | −32.711 to −22.375 | **<0.001** | −26.516 | −31.606 to −21.426 | **<0.001** |

| **n=456** | **Outer macula** | | | | | | | | | | | |
| --- | --- | --- | --- | --- | --- | --- | --- | --- | --- | --- | --- | --- |
|  | **Superior** | | | **Inferior** | | | **Temporal** | | | **Nasal** | | |
| **Predictor variables** | **β** | **95% CI** | ***P* value** | **β** | **95% CI** | ***P* value** | **β** | **95% CI** | ***P* value** | **β** | **95% CI** | ***P* value** |
| Age | −1.057 | −1.863 to −0.250 | **0.010** | −1.191 | −1.925 to −0.456 | **0.002** | −1.309 | −1.988 to −0.630 | **<0.001** | −0.402 | −1.089 to 0.286 | 0.251 |
| Gender | 30.776 | 13.590 to 47.962 | **<0.001** | 28.401 | 12.757 to 44.045 | **<0.001** | 32.166 | 17.702 to 46.630 | **<0.001** | 16.435 | 1.822 to 31.049 | **0.028** |
| Axial length | −24.160 | −29.627 to −18.692 | **<0.001** | −28.256 | −33.281 to −23.231 | **<0.001** | −24.404 | −29.091 to −19.717 | **<0.001** | −22.651 | −27.126 to −18.177 | **<0.001** |

n = number of eyes; ETDRS = Early Treatment Diabetic Retinopathy Study; CI = confidence interval.

β denotes the change in choroidal thickness per unit change in predictor variable.
*P* values in bold indicate statistical significance.
